# A new genus and species of Tullbergiidae (Collembola) from the Pacific Mexican coast

**DOI:** 10.3897/zookeys.326.5451

**Published:** 2013-08-26

**Authors:** José G. Palacios-Vargas, Elihú Catalán

**Affiliations:** 1 Laboratorio de Ecología y Sistemática de Microartrópodos, Departamento de Ecología y Recursos Naturales, Facultad de Ciencias, Universidad Nacional Autónoma de México, 04510 México, D.F.

**Keywords:** Tullbergiidae, Taxonomy, Mexico

## Abstract

The new genus *Mexicaphorura*, and its type species *Mexicaphorura guerrerensis* **sp. n.** are described from marine littoral sand. The new genus is similar to *Sensilatullbergia* Thibaud & Ndiaye, 2006 in having very big sensilla on sensorial organ of antennal segment III, but of different shape, the kind of postantennal organ, with simple vesicles in *Mexicaphorura* versus composed vesicles in *Sensilatullbergia* and formulae of the pseudocelli.

## Introduction

The family Tullbergiidae has 32 genera and 212 species in the word (Bellinger et al. 2013), but from Mexico only 23 species in 6 genera are known (Palacios-Vargas 1997; Thibaud and Palacios-Vargas 2000). It is an euedaphic group of Collembola, which members usually are very small (500–600 µm) without pigmentation, eyes and furcula. The body chaetotaxy of the taxa are very similar, and the main differences among the genera are the sense organ structure of antennal organ III, and the chaetotaxy of the antennal segment IV. The type and number of the pseudocelli and the shape and number of vesicles of postantennal organ have importance at specific and generic level.

Tullbergiidae are much more diverse and abundant in the Southern Hemisphere, where it seems to have its center of dispersion and diversification (Dunger and Schlitt (2011) it has eight endemic genera in this region and only three cosmopolitan genera: *Fissuraphorura*, *Mesaphorura* and *Tullbergia*. While the members of the related family Onychiuridae are widely and often only distributed in the Northern Hemisphere (except for the cosmopolitan genera *Protaphorura*, *Thalassaphorura*, *Agraphorura*, *Deuteraphorura* and *Orthonychiurus*).

Most remarkable contributions on this family from Mexico are those of Bonet (1946) and Palacios-Vargas and Díaz 1995a; 1995b). *Mesaphorura matilei* Thibaud & Palacios-Vargas was described from marine littoral sand (Thibaud and Palacios-Vargas 2000). In this paper we describe one new Tullbergiidae also from marine littoral sand but this is the most extraordinary species found in Mexico because of its morphology.

## Materials and methods

Specimens of the new taxa were obtained by washing sand in sea water and the floating specimens were preserved in alcohol 75%. In the laboratory they were sorted and mounted on Hoyer’s solution for their taxonomic study.

Abbreviations used in this paper are: Ant. antennal segment; Abd. abdominal segment; PAO postantennal organ; Th. thoracic tergite.

## Taxonomy

### Mexicaphorura gen. n.

http://zoobank.org/5194EC74-6F45-4D90-8F3F-797267412A3D

http://species-id.net/wiki/Mexicaphorura

#### Diagnosis.

Habitus of *Mesaphorura*. Small size, about 0.6 mm. Color white. Tegumentary grain fine and uniform. Ant. III with 2 big and modified dorsal sensilla of different shape and one big ventral sensillum cup-like shaped. Formulae of the pseudocelli in the type species 11/122/22221. Pseudocelli of Abd. V guarded by the sensory setae similar to normal setae. Abd. IV tergite without posterior surrounding semicircular narrow ridge. One pair of small and simple anal spines.

#### Type species.

*Mexicaphorura guerrerensis* sp. n.

### Mexicaphorura guerrerensis sp. n.

http://zoobank.org/5BD1D88F-3D9E-477F-8162-1BA4F99A6F13

http://species-id.net/wiki/Mexicaphorura_guerrerensis

Figs 1 –7

#### Material examined.

Type-locality: Mexico: Guerrero State: Marquelia. La Bocana beach. 16°34'60"N, 98°49'0"W; ex littoral marine sand. February and April of 2013, José G. Palacios-Vargas, Jaramar Villareal, Fernando Villagomez collectors. Specimens come from the Pacific Mexican cost, this place is known because the presence of turtle camps for protection of the turtles which come to lay their eggs.

Holotype. Male mounted on slide. Original label: 12/02/2012, ex littoral marine sand from a nest of turtle, J. G. Palacios-Vargas col. Paratypes. 4 females, 2 males, 2 juveniles. Original labels. 14/04/2012, ex litoral marine sand, J. Villarreal and F. Villagomez col. All the material is deposited at Facultad de Ciencias, UNAM.

#### Diagnosis.

Ant. III with 2 big and modified dorsal sensilla of different shape and one big ventral sensillum cup-like shaped. Postantennal organ with 31–43 simple vesicles in four irregular rows. Pseudocelli are star shaped (type 1 of Dunger and Schlitt 2011), formula 11/122/22221. Sensory setae on Abd. V similar to normal setae. Abd. IV without semicircular narrow ridge. One pair of very small and simple anal spines.

#### Description.

Body length (n=9) 600 µm (range 410–720), body with moderately long macrosetae (33 µm), and short microsetae (7µm), all smooth and thin. Tegumentary granules fine, equally distributed on head and thoracic and abdominal tergites, with relatively uniformly distributed fine intergumentary granules, interspersed with somewhat coarser granules on Abd. VI. Antennal bases well delimited.

**Figures 1−3.**
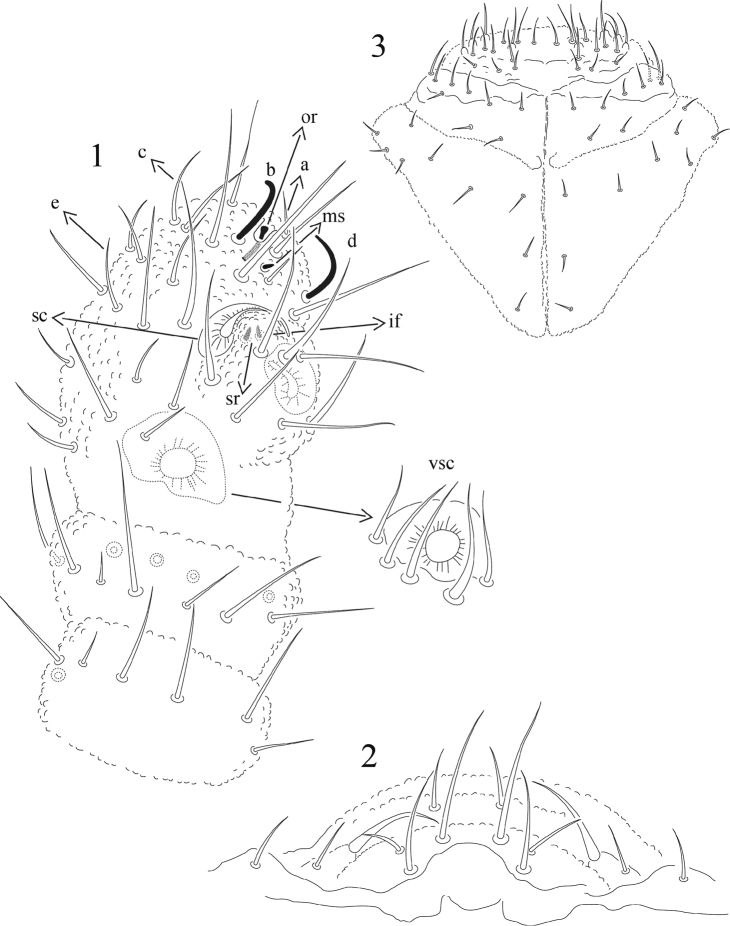
*Mexicaphorura guerrensis* sp. n. **1** dorsal antennal segments I to IV, with detail of the ventral sensillum of Ant. III **2** chaetotaxy of labrum **3** chaetotaxy of labium. a,b,d,d,e = sensilla on Ant. IV, so = subapical organite, ms =microsensillum, sc = thick sensory clubs on Ant. III, sr = sensory rods, if = integumentary fold, vsc = ventral sensory club on Ant. III.

Ratio head: antenna = 1: 0.7. Lengths ratios of Ant. segments I: II; III; IV as 1:0.8; 0.8; 1.3. Sense organ of Ant. III with two small sensory rods concealed behind one big integumentary fold; two thick cup-shaped sensory clubs present, which are rounded at the tip, strongly bend each other and not completely concealed by papillae. Three thick and long guard setae inserted at base of papillae. One big cup-like ventral sensillum protected by five setae (Fig. 1). Ant. IV with 2 thick distinct sensilla, 3 thin sensilla difficult to distinguish except for their size shorter than ordinary setae. There is also one distinct subapical pit with the “organite”, one microsensillum and probably one apical bulb very difficult to distinguish (Fig. 1).

**Figures 4−7.**
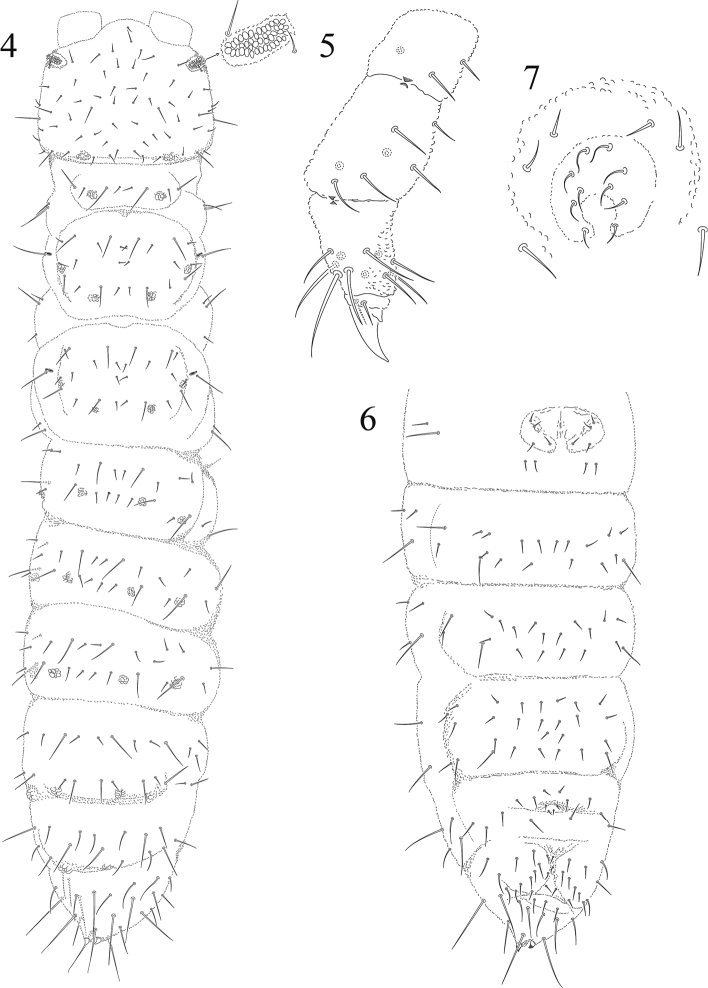
*Mexicaphorura guerrensis* sp. n. **4** dorsal chaetotaxy of body **5** tibiotarsus III **6** ventral abdominal chaetotaxy **7** male genital plate.

Labrum formula, 2/4/2, as illustrated in Fig. 2. Mandible only with one big apical tooth and basal molar surface. Maxilla with six fringed lamellae. Labium with 3 similar pairs of postlabial setae (Fig. 3), and labial palp with one apical thick setae and one thin and short lateral setae (Fig. 2). PAO ovoid, about half as long and the width of the Ant. I, with about 37 (31–43) simple vesicles lying in four irregular rows (Fig. 4).

Leg chaetotaxy from I to III, coxae 3,5,5; trochanters 5,5,5; femora 8,8,8; tibiotarsi 12,12, 11. Claw untoothed. Empodial appendage rudimentary, in shape of a minute claw-like process. Clavate tibiotarsal setae absent (Fig. 5), three dorso-distal setae thicker and longer than the others. Dorsal pseudocelli are irregular in shape and arranged as follows: 11/122/22221 (Fig. 4).

Body chaetotaxy in figure 4 and Table 1. Th. I with one row of 5 + 5 setae, first and third very small, second and fourth long; two lateral setae on each side. Th. II and III with lateral microsensillum, both with 3 rows of setae, m2 absent. Abd. I to Abd. V with 2 rows of setae, Abd. V lacking a2. Pseudocelli are star shaped. Ventral tube with 6 + 6 setae. (Fig. 6). Pseudocelli of Abd. V guarded by sensory setae similar to normal setae. Abd. IV tergite without a surrounding semicircular narrow ridge (Fig. 4).

Female genital plate similar to lips, furnished on upper lip with three pairs of pregenital setae, two circumgenital setae and one pair of eugenital setae (Fig. 6). Male genital plate with two pairs of pregenital setae, two pairs of circumgenital and three pairs of eugenital setae. Anal spines very short and weak, but usually slightly curved and placed on small papillae which touch at their bases (Fig. 4). Ratio anal spine:ungues: 1:1.9.

**Table 1.** Dorsal chaetotaxy of *Mexicaphorura guerrerensis* sp. n.

**row**	**Thorax**			**Abdomen**				
	**I**	**II**	**III**	**I**	**II**	**III**	**IV**	**V**
a	-	10	10	10	10	10	10	8
m	-	8*	8*	2**	2**	2**		8
p	8	10	10	10	10	10	10	8
pl	2	3	2	2	2	3	4	2

* Missing m2,

** m4 present

#### Etymology.

The species is named after the State of Guerrero, where it was found.

#### Distribution.

Known only from the type locality, at Guerrero State, México.

#### Ecology.

This species lives in marine littoral sand.The first specimen was obtained in the floating foam of samples of sand taken from a nest of the Golf turtle *Lepidochelys olivacea* (Cheloniidae). Other specimens were obtained from 12 different places where 4 samples of sand were taken at each and washed. It seems to be very rare species as only 9 specimens were obtained from a total of 48 samples of sand. The new species have fringed maxillae as most of marine littoral Collembola.

#### Discussion.

The new genus is similar *Sensilatullbergia* Thibaud & Ndiaye, 2006 from Senegal because the presence of three big sensilla on Ant. III, two dorsal and one ventral. Because of the number of sensilla on the sensorial organ of Ant. III, this genus belongs to the Tullberginae. Although both genera share the presence of big sensilla on Ant. III, they completely differ in their shape. Other important differences are, vesicles of the postantennal organ in *Mexicaphorura* gen. n. are simple while in *Sensilatullbergia* they are composed of several vesicles each; the number of sensilla on Ant. IV (5 versus 6), apical bulb of antennal segment (indistinguishable versus present), and the sensillum of Abd. V (seta-like versus sensilliform).

*Mexicaphorura guerrerensis* sp. n. is completely different from any Mexican Tullbergiidae known from Mexico. It only can be compared with *Sensilatullbergia senegalensis* Thibaud & Ndiaye, 2006 from littoral marine sand of Senegal because the presence of three big sensilla on Ant. III. Any way they have many differences as the kind of vesicle of the postantennal organ, the number of sensilla on Ant. IV, apical bulb of antennal segment and the sensillum of Abd. V. The chaetotaxy also differs, because the Th. II and III of *Mexicaphorura guerrerensis* sp. n. lacks the seta m2; Abd. V has only two rows of setae while *Sensilatullbergia senegalensis* has 3 rows of setae and the presence of a2.

#### Variation.

The number of vesicles of postantennal organ varies from 31 to 41. The length of PAO varies from 8 to 15 µm. The total body goes from 411 to 670 µm.

## Supplementary Material

XML Treatment for Mexicaphorura

XML Treatment for Mexicaphorura guerrerensis

## Appendix 1

Plate 1

## References

[B1] Bellinger PF Christiansen KA Janssens F (2013) Checklist of the Collembola of the World. http://www.collembola.org

[B2] Bonet F (1946) Tullgerginos de México (Collembola). Revista de la Sociedad Mexicana de Historia Natural 5(1–2): 51−72

[B3] Dunger W Schlitt B (2011) Synopses on Palaearctic Collembola:Tullbergiidae. Soil Organisms 83(1): 1−168

[B4] Palacios-Vargas JG (1997) Catálogo de los Collembola de México.Facultad de Ciencias, UNAM.México, D.F. 102 pp + 10 pls.

[B5] Palacios-Vargas JG Díaz M (1995a) Seven new species of Onychiuridae (Collembola) from the Neotropical Region. Folia Entomol. Mex. 95: 1−21

[B6] PalaciosVargas JG Díaz M (1995b) Survey of the Onychiuridae (Collembola) from Neotropical Region. An. Inst. Biol. Ser. Zool. UNAM. 66(2): 165−180

[B7] Thibaud J-M Ndiaye AB (2006) Collemboles interstitiels des sables littoraux du Sénégal. Revue Française d’Entomologie (N.S.) 28(1): 41−48

[B8] Thibaud J-M Palacios-Vargas JG (2000) Une nouvelle espèce de genre *Mesaphorura* des sables littoraux du Mexique. Revue Française d’Entomologie (N.S.) 22(4): 146−148

