# Supplementary figures and images for: A new genus and species of Tullbergiidae (Collembola) from the Pacific Mexican coast
Source: Zookeys. 2013 Aug 26;(326):91–7. doi: 10.3897/zookeys.326.5451 (PMC3764539; doi:10.3897/zookeys.326.5451)

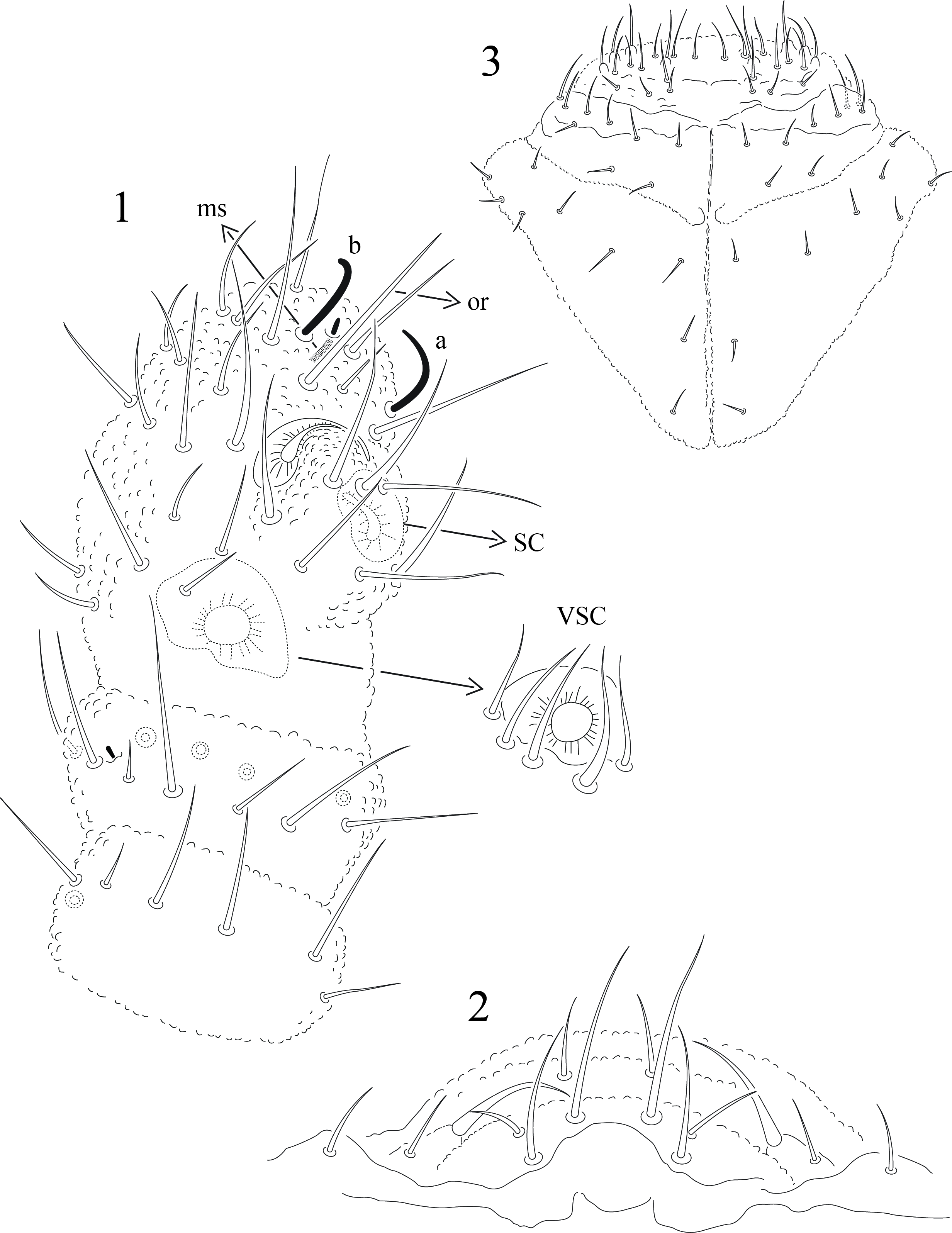

Supplement: Supplementary file 3 — Plate 1 [file ZooKeys-326-091-s001.tif]

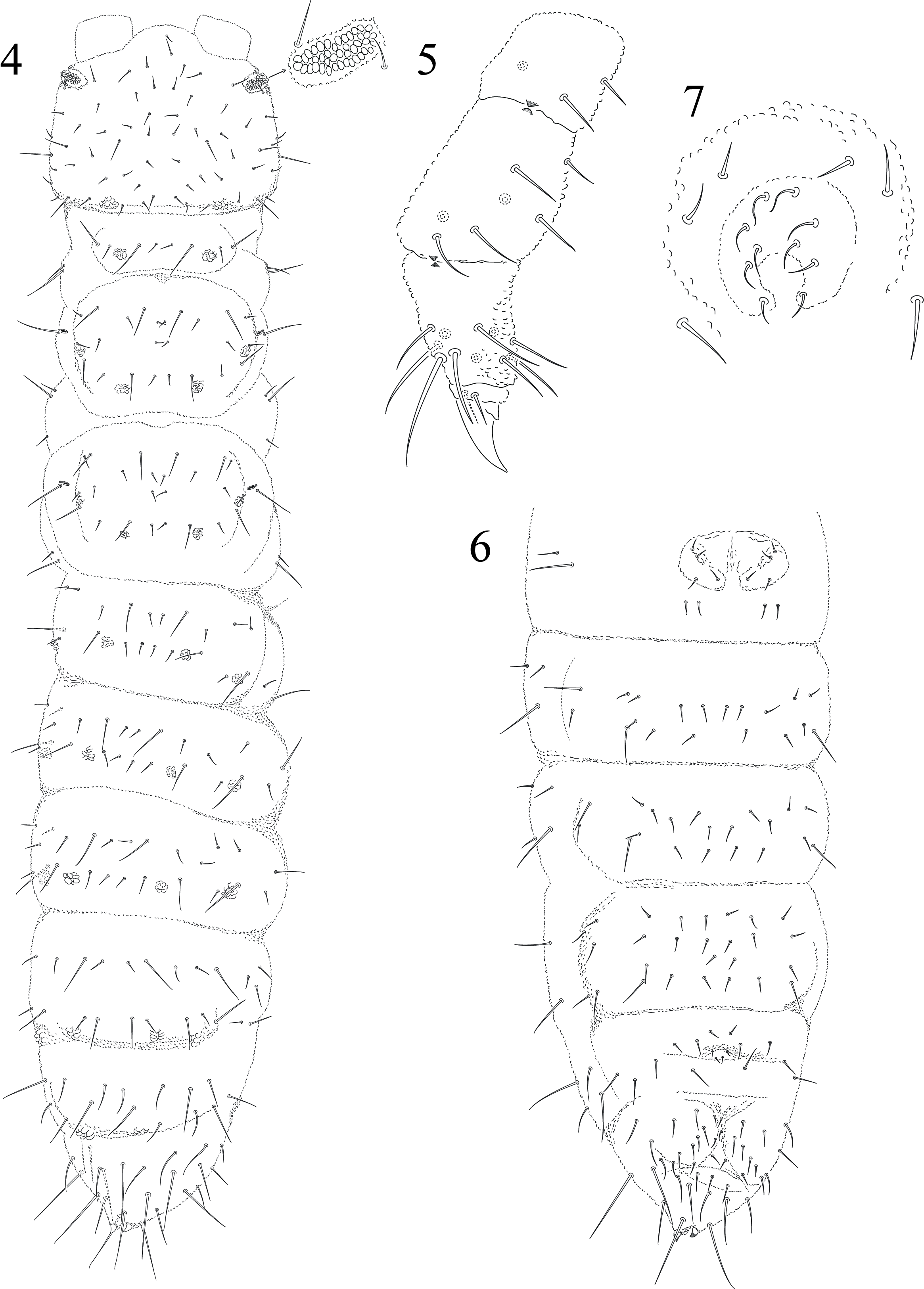

Supplement: Supplementary file 4 [file ZooKeys-326-091-s002.tif]
